# Intraossäre Zugänge bei Säuglingen – Entwicklung eines anatomischen Trainingsmodells

**DOI:** 10.1007/s00063-025-01295-4

**Published:** 2025-06-19

**Authors:** Dietrich Stoevesandt, Lina Woydt, Joachim Koppenberg, Michael Wolf, Dmitrij Pinekenstein, Stefan Watzke, Thomas Lange, Sascha Kolokowsky, Simone Hettmer, Hartmut Stefani, Franz Stangl, Martin R. Fischer

**Affiliations:** 1 Dorothea-Erxleben-Lernzentrum, Medizinische Fakultät, Martin-Luther-Universität Halle-Wittenberg, Magdeburger Straße 12, 06112 Halle (Saale), Deutschland; 2 Institut für Rechtsmedizin, Medizinische Fakultät, Martin-Luther-Universität Halle-Wittenberg, Halle (Saale), Deutschland; 3 Abteilung für Anästhesiologie, Schmerztherapie und Notfallmedizin, Ospidal – Gesundheitszentrum Unterengadin, Scuol, Schweiz; 4 Universitätsklinik und Poliklinik für Psychiatrie, Psychotherapie und Psychosomatik, Martin-Luther-Universität Halle-Wittenberg, Halle (Saale), Deutschland; 5 Klinik und Poliklinik für Pädiatrie I, Universitätsmedizin Halle, Martin-Luther-Universität Halle-Wittenberg, Halle (Saale), Deutschland; 6 Klinik für Notfall- und Akutmedizin, Carl-von-Basedow-Klinikum Saalekreis gGmbH, Merseburg, Deutschland; 7 Klinik für Diagnostische und Interventionelle Radiologie und Neuroradiologie, Universitätsklinikum Augsburg, Augsburg, Deutschland; 8 Institut für Didaktik und Ausbildungsforschung in der Medizin, LMU-Klinikum, LMU München, Deutschland

**Keywords:** Dreidimensionaler Druck, Fehlpunktion, Pediatric Life Support (PLS), Notfallmedikamentengabe, Postmortale Computertomographie, Three-dimensional printing, Malpuncture, Pediatric life support (PLS), Emergency medication, Postmortem computed tomography

## Abstract

**Hintergrund:**

Ein sicherer intraossärer Zugang (i.o.) als Alternative zu einem intravenösen (i.v.-) Zugang ist im Rahmen der Notfallbehandlung von Säuglingen und Kleinkindern essenziell. Die Literatur zeigt jedoch hohe Fehlplatzierungsraten und unzureichende Trainingsmöglichkeiten für potenzielle Anwender.

Ziel der Arbeit war es, Fehlpunktionen in postmortalen computertomografische (CT-) Bildgebungen zu analysieren und ein realistisches, kostengünstiges 3D-gedrucktes Trainingsmodell für i.o.-Punktionen bei Kindern unter 2 Jahren zu entwickeln und zu evaluieren.

**Material und Methoden:**

Computertomografische Daten von 25 verstorbenen Kindern unter 2 Jahren wurden retrospektiv analysiert, um die Häufigkeit und Art von Fehlpunktionen zu dokumentieren. Basierend auf den Erkenntnissen wurde ein 3‑teiliges Modell mittels Filament-3D-Druck und Silikonguss hergestellt. Die realistische Darstellung von Haut, Bindegewebe und Knochen wurde von 55 erfahrenen Anwendern auf einer Likert-Skala evaluiert.

**Ergebnisse:**

Bei 40 % der analysierten Punktionen lag eine Fehlplatzierung vor, häufig bedingt durch eine unzureichende anatomische Einschätzung. Das entwickelte Modell wurde von den Befragten als geeignet für ein Anfängertraining bewertet. Verbesserungsvorschläge betrafen vor allem die Hautkonsistenz und die Simulation des Widerstandsverlustes nach Kortikalisdurchdringung. Die Materialkosten für das Trainingsmodell lagen bei etwa 50 ct pro Punktion.

**Diskussion:**

Das entwickelte 3D-Druck-Modell bietet eine kostengünstige, anatomisch präzise Trainingsmöglichkeit für intraossäre Punktionen bei Säuglingen. Es kann zur Verbesserung der Kompetenz und Sicherheit bei i.o.-Zugängen beitragen, sofern es in regelmäßigen Schulungen angewendet wird. Zukünftige Optimierungen sollten die Hautkonsistenz und den Punktionserfolg weiter realitätsnah simulieren.

**Zusatzmaterial online:**

Zusätzliche Informationen sind in der Online-Version dieses Artikels (10.1007/s00063-025-01295-4) enthalten.

## Abstract (en)

**Background:**

Safe intraosseous (i.o) access as an alternative to intravenous (i.v.) access is essential in the treatment of infants and young children in emergency medicine. However, the literature shows high misplacement rates and insufficient training opportunities for potential users. The aim of this study was to analyze malpunctures in postmortem computed tomography (CT) imaging and to develop and evaluate a realistic, cost-effective three-dimensionally (3D) printed training model for i.o. punctures in children under 2 years of age.

**Materials and methods:**

The CT data from 25 deceased children under 2 years of age were retrospectively analyzed to document the frequency and type of malpunctures. Based on the findings, a three-part model was produced using filament 3D printing and silicone moulding. The realistic representation of skin, connective tissue, and bone was evaluated by 55 experienced users on a Likert scale.

**Results:**

In 40% of the punctures analyzed, there was incorrect placement, often due to inadequate anatomical assessment. The model developed was rated by the interviewees as suitable for beginner training. Suggestions for improvement mainly concerned haptic skin characteristics and the simulation of the loss of resistance after cortical penetration. Material costs for the training model were around 50 cents per puncture.

**Conclusion:**

The 3D printed model offers a cost-effective, anatomically precise training option for intraosseous punctures in infants. It can contribute to the improvement of competence and safety during i.o. access, provided it is supplemented by regular training. Future enhancements should further optimize haptic skin characteristics and should provide better feedback on puncture success.

## Hintergrund und Fragestellung

In der Notfallmedizin stellt ein sicherer Gefäßzugang eine wesentliche Voraussetzung für eine zeitnahe Pharmako- und Volumentherapie Schwerstkranker dar. Intravenöse Zugänge sind insbesondere bei Kindern unter 2 Jahren zeitaufwendiger in der Anlage und weniger häufig erfolgreich als bei älteren Kindern und Jugendlichen [1]. Der intraossäre Zugang gilt in den aktuellen ECR-Leitlinien aus dem Jahr 2021 daher als primärer alternativer Zugangsweg bei Säuglingen und Kindern [2].

In den vergangenen Jahren wurde jedoch eine gehäufte Rate an Fehlplatzierungen von intraossären Zugängen insbesondere bei Säuglingen und Kleinkindern aufgezeigt:

In einer auf postmortaler CT-Bildgebung basierenden Studie wiesen Maxien et al. an 22 Leichen von Kindern unter einem Jahr eine Fehllage von mindestens einer i.o.-Kanüle in 64 % bzw. keinen korrekten Zugang bei 32 % der Kinder nach. In der Altersgruppe über einem Jahr (*n* = 16) wurde bei 50 % mindestens eine Fehllage bzw. bei 19 % keine korrekte Kanülenlage nachgewiesen [3]. Zu ähnlichen Ergebnissen kamen auch Harcke et al. in ihrer Studie an 31 postmortalen Fällen im Alter von 3 Wochen bis 16 Jahren mit 42 tibialen Zugängen. Hier wurden insgesamt 40 % der i.o.-Anlagen als fehlerhaft beschrieben [4]. Pifko et al. fanden bei Kinder ≤ 8 kg miteinander vergleichbare Erfolgsraten von 55 % (17/31) bei manueller Anlage gegenüber 47 % (8/17) bei Nutzung eines Arrow EZ-IO-Bohrsystems (Teleflex, Wayne, Pennsylvania, Vereinigte Staaten) [5]. Andere Studien an Simulationsmodellen zeigten einen Unterschied des Punktionserfolgs abhängig vom verwendeten Punktionsdevice [6, 7]. Außerdem konnte festgestellt werden, dass eine intraossäre Medikamentengabe prähospital zu einer schlechteren Überlebensrate führte [8]. Dies könnte jedoch analog zu Untersuchungen an Traumapatienten zumindest teilweise auf die häufigere Verwendung von i.o.-Zugängen bei schweren Verletzungen zurückzuführen sein [9]. Die meisten publizierten Erfolgsraten liegen damit deutlich unter den Selbstauskünften der German Pediatric Surveillance Unit (GPSU) mit einer Gesamterfolgsrate bei Kindern bis zum 18. Lebensjahr von 98,3 % und einer Erfolgsrate von 81,9 % im ersten Versuch, bei Subanalyse von Kindern unter 1 Jahre mit einer Erfolgsrate von 75,1 % im ersten Versuch. [10]. Dem dort von Pfeiffer et al. diskutierten Underreporting steht in den postmortal durchgeführten Analysen [3, 4] möglicherweise ein negativer Selektionsbias gegenüber. Dieser wird von Harcke et al. ausführlich diskutiert [4].

Die in den ERC-Leitlinien geforderten regelmäßigen Trainings für alle Advanced-Life-Support(ALS)-Provider [2] erscheinen vor dem Hintergrund der veröffentlichten Komplikationsraten umso wichtiger. Eine Vielzahl von Möglichkeiten für i.o.-Trainingsmodelle wurde postuliert und verglichen, wobei sowohl Truthahn- oder Hähnchenbeine als auch kommerziell verfügbare Plastikbeine zwar eine ähnliche Punktionsechtheit bieten [11], jedoch keine dieser Alternativen der durch die Wachstumsfugen und Epiphysenkerne geschuldeten komplexen Anatomie gerecht wird. Ein erster Ansatz der Erstellung eines Punktionsmodells mittels 3D-Druck-Pads wurde 2022 publiziert, allerdings nur mit 12 Personen evaluiert. Dieses Modell stellte außerdem die topografische Anatomie des Säuglingsbeins nicht dar [12]. Das von Manshadi et al. publizierte 3D-gedruckte Modell evaluierte nur den Knochen eines 5‑Jährigen und stellte die umgebende Anatomie (Patella, Haut, Weichteile) nicht da [13].

Dreidimensionaler (3D-)Druck findet in der Medizin bereits eine Vielzahl von Anwendungen. Neben präoperativen Planungen in verschiedenen chirurgischen [14–17] und interventionellen [18, 19] Disziplinen spielt 3D-Druck auch eine zunehmende Rolle in der studentischen Ausbildung [20, 21]. Insbesondere für die Herstellung anatomischer Modelle scheint der 3D-Druck einen Vorteil gegenüber anderen Methoden aufzuweisen [22, 23] und zudem preiswert in der Erstellung [24] zu sein. Dies gilt insbesondere, da der 3D-Druck zunehmend verfügbar ist [25] und basierend auf CT-Daten eine zuverlässige Genauigkeit für die Darstellung von Knochen hat [26].

Die vorliegende Studie soll 3 Fragestellungen klären:

1. In welchem Umfang finden sich i.o-Fehlpunktionen bei Kindern unter 2 Jahren in postmortalen Daten?
2. Ist es möglich, ein kostengünstiges realistisches Punktionsmodell mittels 3D-Druck zu erstellen?
3. Welche technischen Anforderungen muss dieses Modell aufweisen, damit ein für den erfahrenen Anwender hoher Realitätsgrad in Optik, Haptik und Punktionsverhalten generiert wird und das Modell zur Ausbildung von Anfängern ausreichend Feedback zur Erkennung von korrekten und inkorrekten Punktionen gibt?

## Studiendesign und Untersuchungsmethoden

Zur Häufigkeit und Art der Fehllage wurden anonym exportierte CT-Daten der postmortalen CT aller Kinder zwischen 0 und < 2 Jahre in den Jahren 2011 bis einschließlich 2023 der Rechtsmedizin Halle (Saale) analysiert und die Art der intraossären Zugänge (Anzahl, Lokalisation, Auftreten und Art einer Fehllage) dokumentiert. Es wurde zusätzlich systematisch nach typischen Punktionsveränderungen in Tibia und Humerus gesucht und bei Vorliegen ebenfalls Lage und Hinweise auf eine Fehllage dokumentiert. Die dabei verwendeten Datensätze hatten ein auf die Körpergröße adaptiertes Field of View (FOV), die Rekonstruktion erfolgte mit 0,6 mm und einem Inkrement von 0,5 mm. Es wurden der Ort der Punktion und die Art der Fehllage (Perforation der dorsalen Kortikalis, Lage in den Weichteilen ohne Knochenkontakt sowie die Lage in oder distal der Epiphysenfuge, die Lage im Tibiaschaft oder eine intraartikuläre Lage) dokumentiert. Die Datenanalyse erfolgte durch einen Facharzt für diagnostische Radiologie mit 20 Jahren Erfahrung in der postmortalen Bildgebung.

In einem zweiten Schritt wurden aus der CT eines 6 Monate alten Säuglings die DICOM-Daten des rechten Beins extrahiert und mittels der Software Osirix MD (Version 10.0.5, Pixmeo SARL, Bernex, Schweiz) zwei 3D-Formen im SSD-Format erstellt und in ein Standard-Tessellation-Language(stl)-Format exportiert. Eine 3D-Datei entsprach dabei dem bereits verknöcherten Skelett, die andere der Oberfläche des Beins ab Mitte Oberschenkel.

**Abb. 1:**
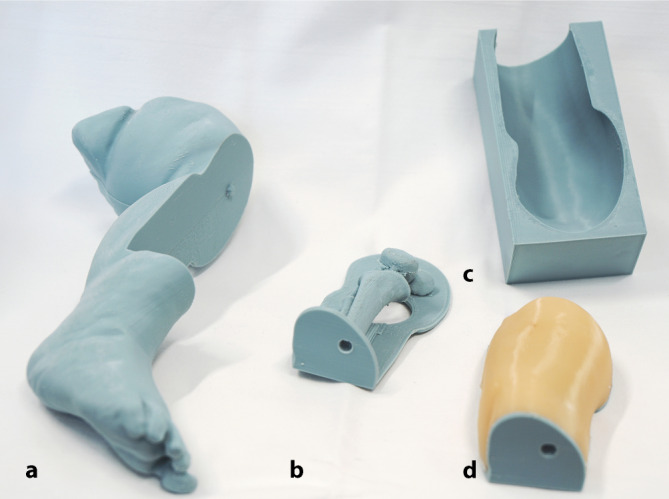
Mittels 3D-Druck erstellte Halterung mit topografischer Anatomie eines 6 Monate alten Säuglings (**a**). In die Halterung eingepasstes Knochenmodell (**b**). Gussform (**c**). Um das Knochenmodell gegossener Silikonkörper (**d**)

Mittels Rhino-6-Software (Version 6.35.21222.17001, Robert McNeel & Associates, Seattle, WA, USA) wurde ein Modell aus beiden Vorlagen erstellt, das aus 3 Teilen bestand:

- distaler Oberschenkel mit Kniekehle und distaler Unterschenkel als Halterung aus Plastikfilament (Polylactid, PLA; siehe Abb. 1a),
- Knochen des Knies und der proximalen zwei Dritteln des Unterschenkels (Knochen ergänzt um Stützstrukturen; [siehe Abb. 1b] ebenfalls aus PLA) und
- Gussform, deren Maße der Oberfläche entsprachen (Abb. 1c).

Zum Druck wurde ein Fused-Filament-Fabrication-Verfahren-Drucker (Prusa i3 MK3S+, Prag, Tschechische Republik) verwendet. Mittels Gussform konnte das den Knochen umgebende Bindegewebe mit Silikon (additionsvernetzendes RTV2-Silikonkautschuk mit Shorehärte [ShA] 00) ergänzt werden (Abb. 1d). Die verwendeten Materialien erlauben sonographisch eine klare Unterscheidung von Weichgewebe und Kortikalis. Die finalen stl-Druckdateien des Modells im stl-Format werden auf Anfrage vom korrespondierenden Autor kostenlos (als CC-BY-NC-SA‑4.0‑Lizenz) zur Verfügung gestellt.

Das verwendete Filament und Silikon wurden durch Abstimmungsphasen mit in der Anlage von i.o.-Zugängen bei Kindern erfahrenen Anwendern mehrmals optimiert.

Zur finalen Version wurden 55 erfahrene Anwender (alle Anwender hatten zumindest einmal einen i.o-Zugang bei einem Kind gelegt) an 10 Kliniken mittels Fragebogen zu den Punktionseigenschaften des Materials befragt und gebeten, ihre Einschätzung jeweils auf einer 7‑stufigen Likert-Skala (1 = volle Zustimmung, 7 = starke Ablehnung) zu dokumentieren. Dabei wurde im Einzelnen nach Stichverhalten von Haut, Bindegewebe und Kochen gefragt und die Nutzer gebeten, die Verwendbarkeit des Modells für ein Anfängertraining allgemein zu bewerten. Zusätzlich waren Freitextantworten möglich und Verbesserungsvorschläge konnten frei vermerkt werden. Da die Fragebögen zusammen mit den Pads in den meisten der 10 beteiligten Kliniken und Rettungsdiensten anonym ausgelegt wurden, kann eine Rücklaufquote nicht berechnet werden. Auch die Anzahl der Rückläufe aus den einzelnen Bereichen liegt nicht vor, da die Fragebögen lediglich die Ausbildung der ausführenden Personen, aus Datenschutzgründen aber nicht die Klinikzugehörigkeit erfassten.

## Ergebnisse

### Fehlpunktionen in der postmortalen CT-Untersuchung

Von den insgesamt 25 Kindern unter 2 Jahren, deren Leichen im Rahmen der postmortalen CT-Bildgebung untersucht wurden, waren 16 männlich (64 %).

**Abb. 2:**
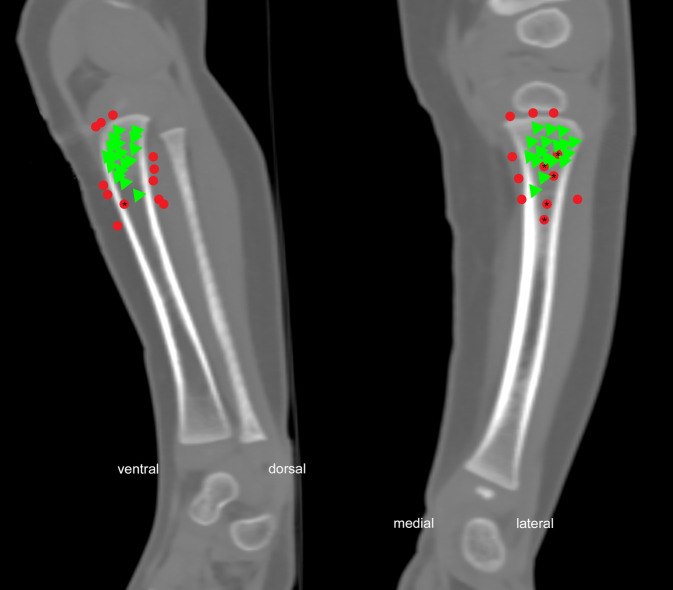
Darstellung aller 29 Punktionen in einem koronaren und sagittalen CT-Schnitt. *Rote Punkte* repräsentieren Fehllagen, *grüne Dreiecke* korrekte Lagen, jeweils bezogen auf das Ende des Stichkanals. Die mit einem *Asterisk* gekennzeichneten Fehlpunktionen liegen aufgrund der Limitation der Darstellung nur scheinbar korrekt, was durch die Visualisierung in der zweiten Ebene offenbar wird

An diesen Kinderleichen konnten 29 Punktionen bzw. Punktionsversuche identifiziert werden (bei 2 Kindern konnten 2, bei einem Kind 3 Punktionsversuche nachgewiesen werden). Eine grafische Übersicht dazu bietet Abb. 2. Bei 17 (59 %) der Punktionsversuche konnte keine Fehllage und kein Paravasat nachgewiesen werden, diese wurden deswegen als erfolgreich gewertet. Unter der Berücksichtigung von mehrmaligen Punktionen bei einem Kind hatten 10 (40 %) der Kinder keinen korrekten i.o.-Zugang. 15 der Punktionen erfolgten an der linken Tibia, 14 an der rechten. Es erfolgte keine Punktion an einer anderen Lokalisation.

**Abb. 3:**
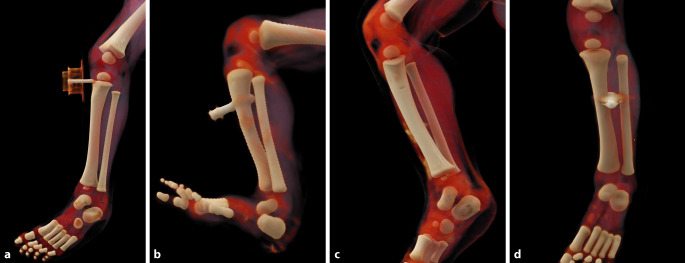
Typische Fehllagen in 3D-Rekonstruktionen aus CT-Daten verstorbener Kindern. **a** 9 Monate alter männlicher Säugling mit Fehllage der Kanüle in der rechten Epiphysenfuge der linken Tibia. **b** Am Tag der Geburt verstorbener weiblicher Säugling mit Perforation der Kanüle durch die dorsale Kortikalis. **c** 6 Monate alter männlicher Säugling mit lateraler Kortikaliskerbe durch i.o.-Kanüle in der linken Tibia. **d** 2 Monate alter männlicher Säugling mit Fehllage zwischen Tibia und Fibula links

Bei 14 Punktionen (48 %) wurde die Kanüle in situ belassen. Von den 12 Fehlpunktionen lagen 3 in der Epiphysenfuge (Abb. 3a), in 2 Fällen wurde die Kortikalis dorsal ein weiteres Mal perforiert (Abb. 3b), 4‑mal wurde die ventrale Kortikalis nicht vollständig perforiert bzw. nur gestreift (Abb. 3c), die übrigen 4 Fehllagen waren komplette Weichteillagen ohne Knochenkontakt (Abb. 3d). Bezogen auf die Höhe der Punktion waren 3 zu weit proximal, 4 waren im Schaft (definiert als > 1 cm unterhalb der Metaphyse) und nicht in der Metaphyse der Tibia lokalisiert. Die Punktion war damit so weit distal, dass sie durch den geringen Schaftdurchmesser erschwert war. Damit waren 58 % der Fehlpunktionen zu weit proximal oder distal. Bezüglich der Abweichungen nach lateral oder medial von einer zentralen Knochenpunktion (unabhängig von der Höhe der Punktion) wichen 5 (42 %) der Fehlpunktionen seitlich ab. Mindestens eine deutliche Abweichung bezüglich der Höhe oder einer lateralen bzw. medialen Abweichung lag bei 10 von 12 Fehlpunktionen (83 %) vor, eine weitere wich vom Einstichwinkel stark vom Knochenzentrum ab.

### Evaluation des Punktionsmodells

Fragebögen zur Qualität des neu entwickelten Trainingsmodells, die von mit der Punktion an Kindern erfahren Personen vollständig ausgefüllt wurden, lagen in 55 Fällen vor (Tab. 1). Auf der 7‑stufigen Likert-Skala schnitt die Bewertung der Haut mit einem Durchschnittswert von 3,3 (SD 1,17) am schlechtesten ab bezüglich der realistischen Darstellung der anatomischen Strukturen. Dabei wurde in den Freitextantworten zur Haut am häufigsten als Änderungswunsch eine derbere Haut (15 Antworten) genannt. Das Füllmaterial wurde mit einem Durchschnittswert 2,4 (SD 1,27) besser bewertet, es gab 5 konkrete Verbesserungsvorschläge, wobei diese teilweise mit „zu weich“ und der „Bitte, es etwas weicher zu gestalten“ gegenläufig waren. Der Knochen wurde von den 3 angefragten anatomischen Kompartimenten mit 2,1 (SD 1,08) am realistischsten bewertet. 2 der 3 konkreten Verbesserungsvorschläge sprachen sich für einen „loss of resistance“ nach Durchstich der Kortikalis aus. Insgesamt bewerteten die Befragten das Modell als geeignet, um Anfänger auf die Punktion vorzubereiten. Auf der 7‑stufigen Likert-Skala lag der Durchschnittswert bei 1,9 (SD 1,17). Als Gesamtverbesserungsvorschläge wünschten sich jeweils eine Person „ein bewegliches Kniegelenk“, „Silikon mit einer erhöhten Haltbarkeit“, „eine direkte Rückmeldung zum Punktionserfolg“, „weitere Modelle in unterschiedlichen Wachstumsphasen“ sowie „eine verbesserte Tastbarkeit von Patella und Gelenkspalt“.

**Tab. 1:** Evaluationsergebnisse des Punktionsmodells durch erfahrene Anwender (*n* = 55) im Überblick auf einer 7‑stufigen Likert-Skala (1 = stimme voll zu, 7 = stimme gar nicht zu)

Parameter	Ø	SD	Änderungswünsche (Anzahl)
*Realitätsnähe der Punktionseigenschaft*			
Haut	3,3	1,17	Derbere Haut (15)
„Füllmaterial“ (Strecke s.c. bis Knochen)	2,4	1,27	Zu fest (2) Zu weich (1) Strecke zu lang (1)
Knochen	2,1	1,08	Zu wenig Widerstandsverlust (2)
*Eignung des Modells*			
Training für Anfänger	1,9	1,17	Bewegliches Kniegelenk (1) Besser tastbare Patella (1) Tibia breiter (1) Austritt applizierter Flüssigkeit (1)

*Ø* Durchschnitt, *SD* Standardabweichung

### Kostenanalyse des Punktionsmodells

Sofern ein einfacher, kommerziell verfügbarer Filamentdrucker vorhanden ist, beliefen sich die Druck- und Stromkosten für die Erstellung einer Beinhalterung (Abb. 1a) einmalig auf etwa 5 €. Diese Halterung konnte mehrmals verwendet werden. Für das Verbrauchsmaterial, also das Punktionspad aus Filamentknochen und Silikon, entstanden Kosten von derzeit 7 €. An einem Pad konnten bis zum Verlust der Silikonstruktur 15–20 Punktionen problemlos durchgeführt werden. Damit entstanden pro Punktion Materialkosten von etwa 50 ct. Für 5 Pads, die parallel hergestellt werden konnten, muss zusätzlich etwa 1 h Arbeitszeit inklusive Vorbereitung und Nachbearbeitung berücksichtigt werden. Für den Druck einer Beinhalterung entstanden etwa 15 min Arbeitszeit. Für die Herstellung reichen einfache 3D-Drucker aus, diese sind ab etwa 200 € verfügbar, der für die Erstellung verwendete Drucker ist für etwa 1000 € erhältlich. Mittels einer orientierenden Internetrecherche wurden 11 kommerziell erhältliche Modelle zur Simulation intraossärer Punktionen bei Kindern mit ausreichend verfügbaren Produktinformationen und Preisen identifiziert. Die Preise lagen zwischen 150 und 2400 € (Stand Februar 2025). In den meisten Fällen wird jedoch ein einfacher Kunststoffstab anstelle eines anatomisch korrekten Knochenmodells verwendet.

## Diskussion

### Fehlpunktionen an Kindern unter 2 Jahren

Durch die geringere Größe des Tibiaplateaus bei Kindern unter 2 Jahren ist ein schlechteres Punktionsergebnis als bei älteren Kindern zu erwarten. Die Kenntnis der genauen anatomischen Verhältnisse hat aus diesem Grund hier wahrscheinlich einen größeren Einfluss, weshalb in der vorliegenden Studie nur Kinder unter 2 Jahren eingeschlossen wurden. Damit ergibt sich ein anderes Kollektiv als in den bisher ebenfalls an Leichen durchgeführten Untersuchungen zur Fehllage von i.o.-Kanülen [3, 4]. Hier finden sich in der vergleichbaren Altersgruppe unter 2 Jahren ähnlich hohe Fehlpunktionsraten von 33 %. In unserem Kollektiv konnten die meisten Fehllagen zumindest teilweise auf Fehler bei der Einschätzung der anatomischen Situation zurückgeführt werden. Auch in der von Harcke et al. durchgeführten Studie können mindestens 30 % der Fehlplatzierungen (5 von 17 Fehllagen, die oberhalb der Epiphysenfuge platziert waren) auf eine Fehleinschätzung der anatomischen Situation zurückgeführt werden. Auch in einer Analyse von Röntgenbildern und der darin erfolgten virtuellen Einpassung von typischen i.o.-Kanülenlängen stellten Capobianco et al. fest, dass bis zu 10,5 % der Kanülen die Markhöhle in Ein- und 18,5 % in 2‑Finger-Breite distal der Tuberositas tibiae nicht erreichen. Die gegenüberliegende Kortikalis wurde in 16 % bzw. 25 % der Fälle berührt oder durchstoßen. Die Autoren folgerten daraus unter anderem, dass die wachstumsbedingten Veränderungen der Tibiaanatomie bei der i.o.-Kanülenanlage berücksichtig werden müssen [27].

### Verbesserungsmöglichkeiten der Trainingsmodelle

Die Mehrzahl der verfügbaren Trainingsmodelle reicht nicht, um Anfänger auf die besonderen, aber für korrekte Positionierung der Kanülen relevanten anatomischen Gegebenheiten am Tibiaplateau vorzubereiten.

Perkins et al. [2] fordern, dass alle pädiatrischen ALS-Anbieter in der Platzierung von i.o-Kanülen kompetent sein und regelmäßig Schulungen zu den verschiedenen Geräten (und Punktionsstellen), die in ihrem Umfeld verwendet werden, absolvieren sollen. In einer Befragung von Pflegepersonal in slowenischen Notaufnahmen gaben jedoch nur 45,3 % der Befragten an, jemals einen intraossären Zugang verwendet zu haben. Nur 74,5 % gaben an, ein Training zur Anlage von i.o.-Kanülen durchlaufen zu haben, davon bewerteten 43,4 % das angebotene Training als insuffizient [28].

Das vorliegende Trainingsmodell ist hygienisch unbedenklich, anatomisch weitestgehend korrekt und kostengünstig. Es kann mit heute einfach verfügbarem 3D-Druck und Gussverfahren dezentral erstellt werden. Aus der Analyse der Befragungsergebnisse und Anwendungserfahrungen aus Kursen ergeben sich folgende mögliche Weiterentwicklungen: festere Haut, Widerstandsverlust nach Durchbohren der Kortikalis, Feedback zum Punktionserfolg mittels Flüssigkeitsaustritt, verbesserte Knochentastbarkeit.

Eine zusätzliche Simulation der Haut (und von Knorpelanteilen) wurde auch von Wade et al. [12] vorgeschlagen. Dabei ließen sich Veränderungen der Hauteigenschaften wahrscheinlich durch ein weiteres, festeres Silikon als erste dünne Schicht in der Gussform erzielen. Dies würde jedoch zu einer komplizierten und teureren Fertigung führen.

Bei einer Punktion am echten Menschen kommt es nach Durchdringen der Kortikalis zu einem Widerstandsverlust, ein wichtiges Feedback zum Punktionserfolg. Dies könnte realisiert werden durch eine dünnere Kortikalis und einen vollständig hohlen Knochen, ohne die aktuell verwendete wabenförmige Füllung.

Ein objektives Feedback zum Punktionserfolg ist didaktisch sinnvoll und könnte durch den direkten Austritt der applizierten Flüssigkeit realisiert werden. Dies wird zwar nur in einem Fragebogen gefordert. Dies wurde auch in mit Studierenden durchgeführten Trainings in einigen Fällen von den Lernenden angesprochen.

Die in einem Fragebogen geforderte verbesserte Tastbarkeit von Knochenstrukturen widerspricht einem der Ziele des Modells, die gerade bei Säuglingen in dieser Hinsicht erschwerten Bedingungen korrekt zu simulieren. In der Optimierungsphase des Modells wurde eine eingeschränkte Tastbarkeit von den befragten Pädiatern zudem expliziert gewünscht.

### Kostenanalyse des Punktionsmodells

Im Vergleich zu den kommerziell verfügbaren Modellen ergibt sich ein deutlicher Kostenvorteil. Manshadi et al. kommen in einer Studie zu 3D-gedruckten Modellen im Vergleich zu einem Hühnchenbeinmodell zu ähnlichen Kosten [13].

Durch die zur Verfügung gestellten Druckdateien kann das hier vorgestellte Modell außerdem nach eigenen Bedürfnissen modifiziert werden.

### Limitationen

Für die postmortal durchgeführten CT-Untersuchungen ist, wie bereits in der Einleitung dargelegt, ein negativer Selektionsbias anzunehmen [4]. Ob sich das Modell zum praktischen Training der i.o.-Punktion eignet, beruht auf der subjektiven Bewertung erfahrener Anwender. Inwieweit ein Training an diesem Modell tatsächlich zu einer Verbesserung des Punktionserfolgs beiträgt, kann daher anhand der vorliegenden Studie nicht zuverlässig beurteilt werden.

### Schlussfolgerung und Ausblick

Um die hohen Fehlpunktionsraten bei Kindern unter 2 Jahren zu reduzieren, wird gefordert, i.o. Punktionen regelmäßig zu trainieren. Das vorgelegte Modell eignet sich insbesondere für Anfänger dazu, da es die anatomischen Verhältnisse realitätsnah abbildet. Als 3D-Druck ist es kostengünstig und niedrigschwellig selbst herstellbar. Eine Herstellungsanleitung findet sich im Supplement (Supplement 1). Die Weiterentwicklung des Modells ist notwendig und kann je nach geplantem Einsatz variieren. Anpassungen sind unter der Creative-Commons-Lizenz (CC BY-NC-SA 4.0) auch für andere Nutzer möglich.

Das erstellte Modell kann in praktischen Kursen zur i.o-Punktion verwendet werden, diese werden aktuell konzipiert. Sie sollten hinsichtlich ihrer Eignung zur Verbesserung des Punktionserfolgs evaluiert werden.

## Fazit für die Praxis

- Fehlpunktionen bei intraossären Zugängen sind bei Kindern unter 2 Jahren häufig und in der Regel durch anatomische Fehlplatzierung bedingt.
- Ein anatomisch korrektes Punktionsmodell lässt sich mit gut verfügbaren Techniken preiswert herstellen.

## Supplementary Information

Supplement 1 Herstellung i.o. Punktionstrainer

Beinhalterung (.stl)

Knochenbasis für Silikonpad (.stl)

Gussform für Silikonpad (.stl)

## Data Availability

Alle Daten, inclusive der verwendeten stl-Druckdateien, sind auf Anfrage beim korrespondierenden Autor verfügbar.
