# Supplementary material for: Intraosseous access in infants—development of an anatomical training model
Source: Med Klin Intensivmed Notfmed. 2025 Jun 19;121(3):201–7. [Article in German] doi: 10.1007/s00063-025-01295-4 (PMC13038734; doi:10.1007/s00063-025-01295-4)

## Herstellung i.o. Punktionstrainer

Der Punktionstrainer für einen intraossären Zugang bei Säuglingen und Kleinkindern umfasst 1. eine wiederverwendbare Grundform für das Bein sowie 2. ein Wechselpad.

### Bein:

Die Druckdateien wurden unter Zuhilfenahme eines CT-Datensatzes generiert, wobei sowohl knöcherne als auch weichteilige Anteile berücksichtigt wurden.

Die Grundform des Beines wird aus weißem oder hautfarbenem PLA gefertigt.

Das Bein-Modell wird mit einer Schichthöhe von 0,2 mm, einem Infill von 15 % und vier Perimetern (Außenlinien) auf einem Prusa MKS3 gedruckt.


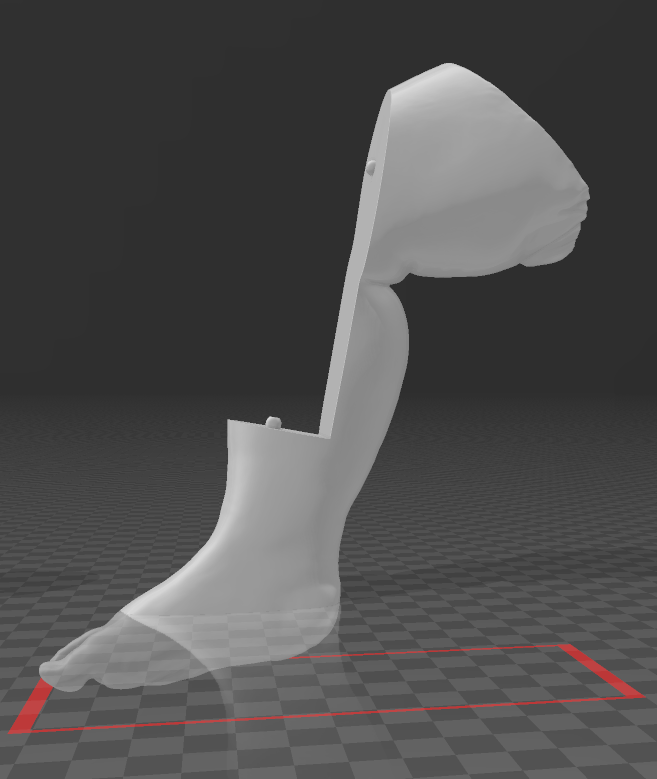


### Wechselpad:

Für die Erstellung der Druckdatei des Wechselpads wurden primär die Datensätze der relevanten knöchernen Anteile herangezogen. Der knöcherne Anteil des Wechselpads wird ebenfalls aus PLA gedruckt, wobei eine Schichthöhe von 0,2 mm, ein Infill von 20 % sowie vier Perimeter (Außenschichten und obere Schichten) verwendet werden.

Das umliegende Gewebe wird unter Zuhilfenahme einer Gussform und Silikonkautschuk hergestellt.

Die Shorehärte 00 des Silikonkautschuks entspricht in etwa der haptischen Festigkeit menschlichen Gewebes. Durch die Zugabe spezieller Pigmente kann ein opakes Punktionsmodell erzeugt werden.

Die Verarbeitungszeit des verwendeten Silikonkautschuks beträgt ca. 20 min, während die Aushärtungszeit 6 h in Anspruch nimmt.

Das Punktionspad kann ca. 20-25 mal punktiert werden bevor es zu einem spürbaren Verlust des Punktionswiederstandes kommt.


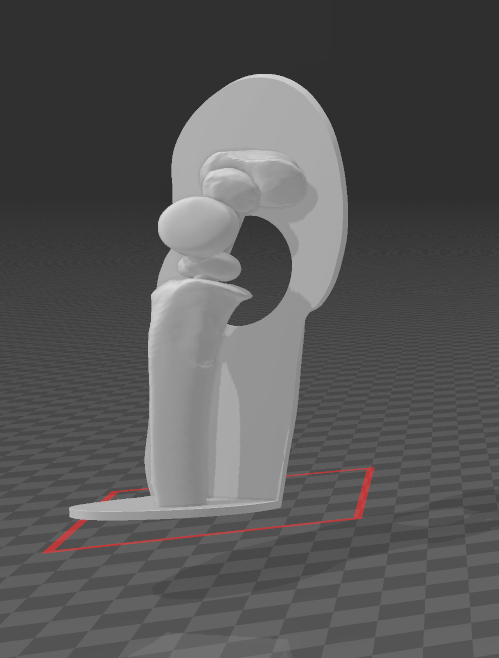


### Die Gussform

Die Gussform wurde durch Subtraktion der Oberflächenform des betreffenden Beinabschnittes, d. h. des Wechselpads, von einem virtuellen Quader erzeugt. Die Schichthöhe der Gussform wurde auf 0,1 mm festgelegt, um eine feine Oberflächenstruktur zu erzeugen. Das Infill beträgt 30 % und die Anzahl der Außenhüllen beläuft sich auf vier. Im Anschluss an den Druck wird die Oberfläche der Gussform leicht angeschliffen, um beim Guss eine möglichst glatte Oberfläche zu gewährleisten. Vor dem Guss wird die Oberfläche mit Trennspray behandelt, um das fertige Wechselpad leichter aus der Form lösen zu können. In die so entstandene Form wird der knöcherne Anteil des Wechselpads eingeklickt und der Hohlraum aufgegossen, wodurch ein Punktionsmodell mit realistischer Gewebedicke und korrekten anatomischen Landmarken entsteht.


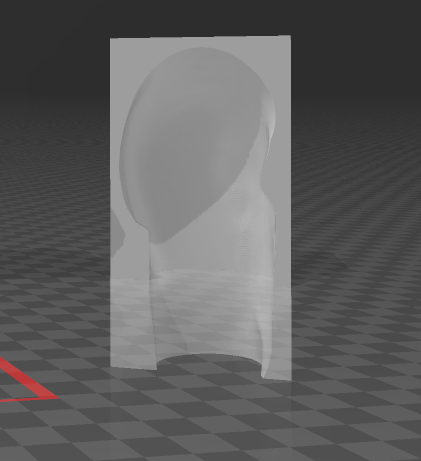

Supplement: Supplementary file 1 — Supplement 1 Herstellung i.o. Punktionstrainer [file 63_2025_1295_MOESM1_ESM.docx]
